# Identification of the raw and processed *Crataegi* Fructus based on the electronic nose coupled with chemometric methods

**DOI:** 10.1038/s41598-020-79717-w

**Published:** 2021-01-20

**Authors:** Chenghao Fei, Chenchen Ren, Yulin Wang, Lin Li, Weidong Li, Fangzhou Yin, Tulin Lu, Wu Yin

**Affiliations:** 1 School of Pharmacy, Nanjing University of Chinese Medicine, Nanjing, China; 2 State Key Lab of Pharmaceutical Biotechnology, College of Life Sciences, Nanjing University, Nanjing, China

**Keywords:** Chemical biology, Plant sciences

## Abstract

*Crataegi* Fructus (CF) is widely used as a medicinal and edible material around the world. Currently, different types of processed CF products are commonly found in the market. Quality evaluation of them mainly relies on chemical content determination, which is time and money consuming. To rapidly and nondestructively discriminate different types of processed CF products, an electronic nose coupled with chemometrics was developed. The odour detection method of CF was first established by single-factor investigation. Then, the sensor array was optimised by a stepwise discriminant analysis (SDA) and analysis of variance (ANOVA). Based on the best-optimised sensor array, the digital and mode standard were established, realizing the odour quality control of samples. Meanwhile, mathematical prediction models including the discriminant formula and back-propagation neural network (BPNN) model exhibited good evaluation with a high accuracy rate. These results suggest that the developed electronic nose system could be an alternative way for evaluating the odour of different types of processed CF products.

## Introduction

*Crataegi* Fructus (CF) is obtained from the plants of the genus *Crataegus* of the Rosaceae family, which is mainly distributed in the Northern Hemisphere including Asia, Europe, and North America^1,2^. Although more than 1000 species have been reported worldwide, *Crataegus monogyna* and *C. lavigata* are the major hawthorn species in Europe, and *C. pinnatifida* is the major one in Asia^1^. *Crataegus* leaves and flowers are often used as medicinal and food materials in western countries, but in Asia, it is commonly cultivated for fruits^3^. Many studies have demonstrated that CF from *C. pinnatifida* can decrease blood pressure and hyperlipidaemia by enhancing coronary flow, myocardial contractility, and cardiac output^4–6^. Moreover, it exhibits useful antibacterial and antioxidant activities^7^. Due to excessive acidity, CF ingestion usually leads to increased gastric acid secretion and membrane irritation. To avoid these side effects, CF is often processed for clinical use. Four types of processed CF products are available in the market^8^. Raw *Crataegi* Fructus (RCF), which is freshly cleaned and smells slightly fragrant and sour, can promote blood circulation. RCF is heated at a low temperature (approximately 150 °C) to form Chao *Crataegi* Fructus (CCF), where the sour smell is reduced. Heating RCF at medium (approximately 180 °C) and high (approximately 200 °C) temperatures yields Jiao *Crataegi* Fructus (JCF) and Tan *Crataegi* Fructus (TCF), respectively. After stir-frying, the sour smell of CF decreases gradually, but coke flavour is increasingly strengthened. An increase in heating temperature gradually attenuates CF acidity. Meanwhile, different types of processed CF products have different functions in clinical use; for example, RCF treats hyperlipidaemia^9^ and hypertension^10^, CCF promotes digestion^11^, JCF promotes digestion^12^ and treats diarrhoea^13^, and TCF promotes haemostasis^14^ and treats diarrhoea^15^.

Odour is one of the most important indicators for evaluating the quality of herbal medicines. Every herbal medicine has its own special odour, whether strong or weak, which is directly related to its components. Therefore, the trait and intensity of the odour are probably determined by the authenticity and quality of the herbal medicine. The odour is the comprehensive external expression of the components of herbal medicines. However, it is undeniable that odour detection depends on the human olfactory system, which is influenced by many major factors including the differences of inspector and environment^16^. Thus, the objectivity and accuracy of human olfactory evaluation are difficult to be guaranteed, which limits the use of odour as an indicator in practical applications and long-term promotions. It is necessary to use advanced technology and equipment to objectively quantify the empirical odour expressions of herbal medicines.

The electronic nose (also known as the odour fingerprint technology, E-nose) is an analytical device with the ability to identify pure and mixed gases by multiple performance overlapping gas sensors and appropriate composition of pattern ordination methods^17,18^. The E-nose can be traced back to the 1960s. After 30 years of development, the E-nose was not defined until the 1990s. It is mainly composed of the sampling system, a gas sensor array, a signal processing system, and a pattern recognition system, and can simulate the olfactory sensation of humans and animals to judge and analyse odour characteristics^19,20^. Presently, due to the multiple advantages of being non-invasive, having fast response, ease of use, and low cost, it has been successfully applied in numerous fields including food product quality assessment, environmental monitoring, bio-security, agriculture industry, and medical diagnostics^21–23^. One of the important properties of E-nose is that it can reflect the macroscopic characteristics and classify the quality grade for materials. Since many herbal medicines possess unique odours, E-nose is suitable for evaluating their quality. However, previous studies have mainly focused on the authentication and geoherbalism of different species, which was not sufficient and comprehensive for the quality control of different types of processed herbal medicines.

Conventional CF odour quality control (QC) methods are mainly dependent on human olfaction. Therefore, there is an increasing demand to develop objective QC methods for a rapid analysis of CF odour. In this study, the electronic nose was employed to evaluate the quality of different types of processed CF products. After the detection conditions were established and sensors optimised, the digital and mode standard of odour response were established by percentiles and discriminant factor analysis (DFA), respectively. Additionally, the treatment methods of different types of processed CF products were predicted by the discriminant formula and back-propagation neural network for the first time.

## Results

### Electronic nose response of samples

**Figure 1:**
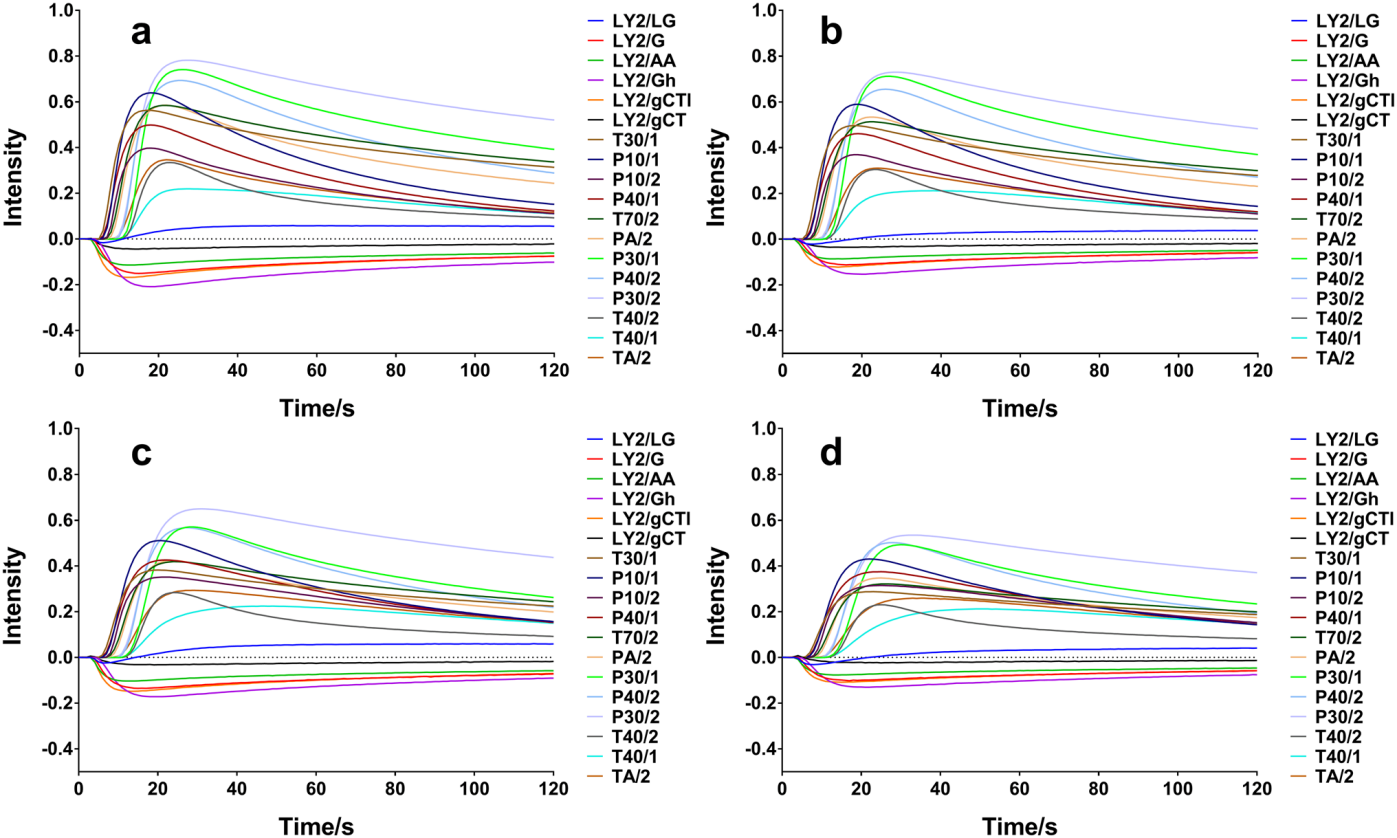
Sensor response curves of the odour of different CF groups. (**a**) RCF; (**b**) CCF; (**c**) JCF; (**d**) TCF.

The typical signal records of the 18 sensors for RCF, CCF, JCF, and TCF are shown in Fig. 1. Each curve represents the conductivity of one sensor induced by the electro-valve action when volatile gas reaches the measurement chamber^24^. The sensor response was calculated by the following equation: *R* = (*R*_0_ − *R*_t_)/*R*_0_, where *R*, *R*_t_, and *R*_0_ represent the sensor response, instantaneous sensor resistance, and sensor resistance at 0 s, respectively. Positive sensor response indicates that the reduction of gas is stronger than oxidation, while a negative response indicates that the oxidation of gas is stronger than reduction. In one test, the sensor resistance was measured every second for 120 s and the data were recorded using the software Alpha Soft 11.0. As shown in Fig. 1a–d, curves of different CF samples show a similar trend. Normally, there is a minimum relative standard deviation (RSD) for peak or valley data of the same sample curve, which is conducive to the maximum identification of different samples. Therefore, the maximum response value for each sensor was extracted and analysed individually.

### Optimisation of the condition for E-nose detection

To obtain the highest response and stability, the main parameters were optimised by single-factor investigation, such as the sample particle size (original size, 10, 24, 50, and 65 mesh), sample quantity (0.1, 0.2, 0.3, 0.4, and 0.5 g), injected volume (500, 800, 1000, 1200, and 1500 µL), headspace temperature (40, 45, 50, and 55 °C) and headspace time (100, 150, 200, 250, 300 and 600 s). The effects of sample treatment conditions and E-nose detecting parameters on the sensor response were shown (Supplementary Fig. S1). The principle of optimisation is to ensure that the response values of most sensors are 0.3 ~ 0.9 and the corresponding RSDs are as small as possible. Most sensors had the optimum response value with lowest response RSD when the sample powder was at size of 50 mesh, the loading amount of sample was 0.5 g, and the injected volume, headspace temperature and time of the examination were set at 1200 µL, 55 °C, and 600 s, respectively.

### Validation of the method for odour detection

The repeatability of the method was evaluated by measuring and analysing the same sample in six parallel tests. The RSD of the response value for each sensor was found to be < 5% (Supplementary Table S1). The sample stability was evaluated by analysing the same sample for different time periods (0, 2, 4, 6, 8, 10, 14, 16 h), and the sample was found to be stable for 16 h with RSDs less than 5% (Supplementary Table S2).

### Optimisation of the sensor array

The data of each sample contained 18 response values of E-nose sensors. Thus, for the original sensor array, named U_0_, a data matrix (18 × 176) was obtained. To explore the correlation and avoid any problems of dimensionality, SDA and ANOVA were employed for data reduction based on the data matrix of U_0_.

#### Optimisation of the sensor array by stepwise discriminant analysis

Wilks’ Lambda method was used for SDA, with *F* values as discriminant statistics. The default values of *F* in this study were: when *F* ≥ 3.84, the variable entered the model; when *F* ≤ 2.71, the variable was moved out of the model. Stepwise selection started with the variable that had the largest *F* value and smallest Wilks’ lambda value. In this study, the procedure ended after 10 steps. A total of 10 sensors were eventually identified by the model, where the *F* and *p* values were > 3.84 and < 0.01, respectively (Supplementary Table S3). Finally, the optimised sensor array obtained by SDA, U_1_, was composed of LY2/AA, LY2/gCTl, T30/1, P10/2, PA/2, P30/1, P40/2, P30/2, T40/2, and T40/1.

#### Optimisation of the sensor array by the analysis of variance

In ANOVA, the discrimination ability of the variable was decided by the *F* value and the *p* value between the groups, and the repeatability of the variable was decided by the mean squares within groups. Thus, sensor contribution to discrimination increased with an increase in *F* value or a decrease in *p* value between groups, and sensor repeatability increased with a decrease in the mean squares within groups. Except for LY2/LG, LY2/G, LY2/AA, LY2/Gh, LY2/gCTl and T40/1, the *p* value of the 12 retained sensors was < 0.01. Furthermore, among the 12 retained sensors, the mean squares of LY2/gCT, P10/2, P40/1, P30/1, P40/2, T40/2 and TA/2 were < 3 × 10^−3^ (Supplementary Table S4). Finally, according to sensor *p* value between groups and mean squares within groups, the optimised sensor array obtained by ANOVA, U_2_, was composed of LY2/gCT, P10/2, P40/1, P30/1, P40/2, T40/2 and TA/2.

#### Comparison of classification before and after sensor array optimisation

**Figure 2:**
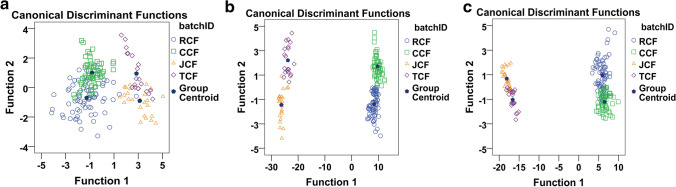
LDA analytical results of CF groups based on different sensor arrays. (**a**) Original sensor array U_0_; (**b**) optimised sensor array U_1_ by SDA; (**c**) optimised sensor array U_2_ by ANOVA_._

To identify the best sensor array for following data analysis, linear discriminant analysis (LDA) was applied to compare the three sensor arrays (U_0_, U_1_, and U_2_) in the projected graph. LDA analytical results of the four processed CF product groups are shown in Fig. 2. Linear discriminant (LD) factors explained 94.6% of the total variance in the dataset of U_0_, with LD1 and LD2 representing 76.8% and 17.8%, respectively, and all the CF groups were overlapped. The total LD1 and LD2 contribution was > 99% of the optimised sensor arrays (U_1_ and U_2_), which implied that the established LD function could explain most of the information. Meanwhile, compared with U_0_, U_1_ and U_2_ intuitively improved CF sample distribution, with almost no overlap.

Furthermore, the classification was digitally evaluated by LDA. The classification value further showed that the optimised array is better than the un-optimised array. As shown in Table 1, the correct classification rate of CF samples for different sensor arrays are as follows: U_1_ (98.9%) > U_2_ (86.4%) > U_0_ (77.8%), and the sensor array U_1_ obtained by SDA had the highest correct classification rate. Therefore, U_1_ was identified as the optimum sensor array and used for further data analysis.

**Table 1:** Results of classification by LDA for three optimised sensor array.

		Group	Predicted group membership				Total	Correct classification rate
			1	2	3	4		
U_0_	Count	1	54	17	0	3	74	77.8%
		2	11	45	0	0	56	
		3	0	0	26	0	26	
		4	0	0	8	12	20	
	%	1	73.0	23.0	0	4.1	100.0	
		2	19.6	80.4	0	0	100.0	
		3	0	0	100.0	0	100.0	
		4	0	0	40.0	60.0	100.0	
U_1_	Count	1	74	0	0	0	74	98.9%
		2	0	56	0	0	56	
		3	0	0	24	2	26	
		4	0	0	0	20	20	
	%	1	100.0	0	0	0	100.0	
		2	0	100.0	0	0	100.0	
		3	0	0	92.3	7.7	100.0	
		4	0	0	0	100.0	100.0	
U_2_	Count	1	57	17	0	0	74	86.4%
		2	2	54	0	0	56	
		3	0	0	23	3	26	
		4	0	0	2	18	20	
	%	1	77.0	23.0	0	0	100.0	
		2	3.6	96.4	0	0	100.0	
		3	0	0	88.5	11.5	100.0	
		4	0	0	10.0	90.0	100.0	

*U*_*0*_ original sensor array, *U*_*1*_ optimized of the sensor array by SDA, *U*_*2*_ optimized of the sensor array by ANOVA.

1, 2, 3, 4 in column: the sample actually belonged to RCF, CCF, JCF, TCF, respectively.

1, 2, 3, 4 in row: the sample was predicted to RCF, CCF, JCF, TCF, respectively.

### Establishment of the digital standard of odour response

The multivariate normality tests of sample data (four CF groups) were conducted. According to the normal distribution of the tested standard (*p* < 0.05), most odour response values do not conform to the normal distribution (Supplementary Table S5). Thus, bilateral 90% confidence intervals were chosen to establish a reference range of odour response of the four CF groups based on U_1_, where the P5 and P95 percentile indices were obtained by the frequency statistics of descriptive analysis. The results are shown in Table 2.

**Table 2:** Odour standard range of different processed products of *Crataegi* Fructus.

Sensor	RCF	CCF	JCF	TCF
LY2/AA	− 0.218 ~ − 0.084	− 0.228 ~ − 0.066	− 0.118 ~ − 0.075	− 0.191 ~ − 0.064
LY2/gCTl	− 0.364 ~ − 0.116	− 0.388 ~ − 0.090	− 0.177 ~ − 0.107	− 0.320 ~ − 0.088
T30/1	0.479 ~ 0.721	0.383 ~ 0.741	0.342 ~ 0.486	0.288 ~ 0.618
P10/2	0.360 ~ 0.486	0.328 ~ 0.501	0.335 ~ 0.412	0.315 ~ 0.479
PA/2	0.514 ~ 0.710	0.439 ~ 0.729	0.393 ~ 0.523	0.348 ~ 0.632
P30/1	0.685 ~ 0.806	0.642 ~ 0.836	0.528 ~ 0.651	0.493 ~ 0.746
P40/2	0.643 ~ 0.801	0.590 ~ 0.814	0.544 ~ 0.644	0.502 ~ 0.736
P30/2	0.721 ~ 0.881	0.625 ~ 0.891	0.614 ~ 0.758	0.535 ~ 0.854
T40/2	0.293 ~ 0.424	0.252 ~ 0.439	0.262 ~ 0.339	0.231 ~ 0.410
T40/1	0.206 ~ 0.315	0.198 ~ 0.330	0.214 ~ 0.242	0.212 ~ 0.284

The 5th and 95th percentiles in the exploratory analysis were selected to build the 90% range.

The rationality of the reference range for processed CF products was verified by the nonparametric test. The method of K Independent Samples, including Kruskal–Wallis H Test and Median Test, was used to analyse the response value from CF samples. The *p* values of the response from 10 sensors were < 0.01, which indicated that the difference in the reference range of the four CF groups was statistically significant (Supplementary Table S6). The identification of unknown samples is based on whether the response of each sensor is within the corresponding reference range.

### Establishment of modelling standard of odour response

**Figure 3:**
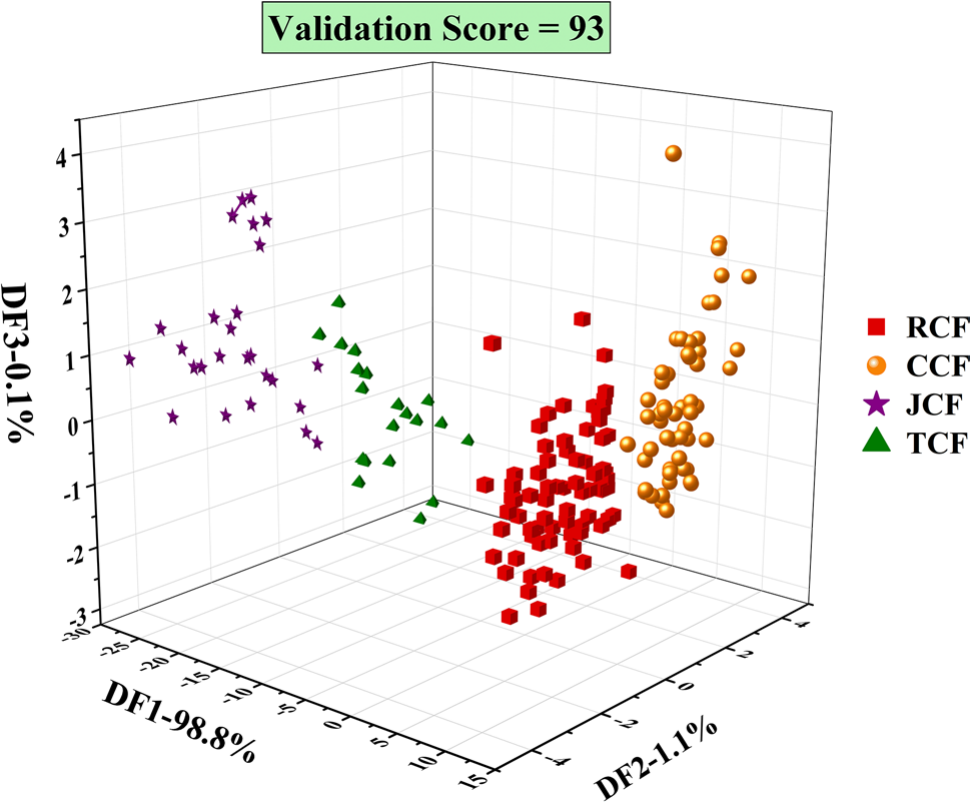
DFA recognition pattern of different CF groups. “DF” is the discriminate function, which is a linear combination (sum) of the discriminating variables. The contribution rates of DF1, DF2 and DF3 are 98.8%, 1.1% and 0.1%, respectively. Validation score is obtained by cross-validation (leave-one-out).

To establish a visual recognition pattern for the rapid determination of unknown samples, DFA was used to build an odour response database based on U_1_. As shown in Fig. 3, the total contribution rate of three-dimensional model reaches 100%, and the contribution rates of DF1, DF2 and DF3 are 98.8%, 1.1% and 0.1%, respectively, which shows that the information of the original samples could be explained by the established model reliably (> 85%). Furthermore, CF samples were divided clearly into four regions, and the cross-validation score of the model was 93, indicating that the model effectively distinguished different types of processed CF samples. The distribution of different CF groups showed certain characteristics. For example, RCF and CCF groups were distributed in the positive direction of DF1, while JCF and TCF groups were distributed in the negative direction. Simultaneously, CCF and TCF groups were distributed in the positive direction of DF2, while RCF and JCF groups were distributed in the negative direction. Different CF groups were distinguished well.

When the unknown sample is input into the discriminant model and projected to a specific area, it will be identified as a corresponding group according to the recognition value (> 70%) or intuitive projection result. Otherwise, the unknown sample will be judged as “unrecognised”.

### Prediction of different processed CF by discriminant formula

To build mathematical functions to predict the processing degree of CF, each sensor response dataset from RCF, CCF, JCF, and TCF, based on the response of U_1_, was analysed using bayesian linear discriminant analysis (BLDA). The odour response values of all samples from the four groups were loaded into SPSS 23.0 to carry out the analysis. The canonical discriminant functions of raw and processed CF built by BLDA are as follows:

$$\begin{aligned} F_{{1}} & = \, - { 1}.{35}0 \times {1}0^{{4}} LY2/G + { 1}.{326} \times {1}0^{{4}} LY2/gC{\text{T}}l \\ & \quad {-}{ 8}.0{75} \times {1}0^{{3}} T30/1{-}{ 6}.{764} \times {1}0^{{3}} P10/2 + { 2}.{778} \times {1}0^{{3}} PA/2 \\ & \quad + { 4}.{7}0{7} \times {1}0^{{3}} P30/1 + { 3}.{564} \times {1}0^{{4}} P40/2{-}{ 8}.{2}00 \times {1}0^{{3}} P30/2 \\ & \quad {-}{ 1}.{746} \times {1}0^{{4}} T40/2 + { 1}.{122} \times {1}0^{{4}} T40/1 - { 6}0{83}.{726,} \\ \end{aligned}$$

$$\begin{aligned} F_{{2}} & = \, - { 1}.{256} \times {1}0^{{4}} LY2/G + { 1}.{288} \times {1}0^{{4}} LY2/gCTl \\ & \quad {-}{ 7}.{846} \times {1}0^{{3}} T30/1{-}{ 6}.{46}0 \times {1}0^{{3}} P10/2 + { 2}.{284} \times {1}0^{{3}} PA/2 \\ & \quad + { 5}.{3}0{9} \times {1}0^{{3}} P30/1 + { 3}.{596} \times {1}0^{{4}} P40/2{-}{ 8}.{743} \times {1}0^{{3}} P30/2 \\ & \quad {-}{ 1}.{743} \times {1}0^{{4}} T40/2 + { 1}.{116} \times {1}0^{{4}} T40/1 - { 6241}.{227,} \\ \end{aligned}$$

$$\begin{aligned} F_{{3}} & = \, - { 2}.{439} \times {1}0^{{4}} LY2/G + { 1}.{799} \times {1}0^{{4}} LY2/gCTl \\ & \quad {-}{ 1}.{344} \times {1}0^{{4}} T30/1{-}{ 1}.{499} \times {1}0^{{3}} P10/2 + { 1}.{138} \times {1}0^{{3}} PA/2 \\ & \quad + { 1}.{867} \times {1}0^{{3}} P30/1 + { 2}.{685} \times {1}0^{{4}} P40/2{-}{ 3}.{877} \times {1}0^{{3}} P30/2 \\ & \quad {-}{ 2}.{848} \times {1}0^{{3}} T40/2 + { 9}.{219} \times {1}0^{{3}} T40/1 - { 4879}.{96}0, \\ \end{aligned}$$

$$\begin{aligned} F_{{4}} & = \, - { 2}.{23}0 \times {1}0^{{4}} LY2/G + { 1}.{7}0{5} \times {1}0^{{4}} LY2/gCTl \\ & \quad {-}{ 1}.{3}00 \times {1}0^{{4}} T30/1{-}{ 8}.0{24} \times {1}0^{{2}} P10/2 + { 8}.{475} \times {1}0^{{2}} PA/2 \\ & \quad + { 2}.{433} \times {1}0^{{3}} P30/1 + { 2}.{754} \times {1}0^{{4}} P40/2{-}{ 4}.{837} \times {1}0^{{3}} P30/2 \\ & \quad - {3}.{366} \times {1}0^{{3}} T40/2 + { 8}.{793} \times {1}0^{{3}} T40/1 - { 4961}.{121}{\text{.}} \\ \end{aligned}$$

When the response value of the unknown sample is substituted into the above discriminant functions to calculate *F*, the processing degree of the unknown sample will be determined according to the highest *F* value. Thus, if the *F*_1_ value is the highest, it is a RCF; if the *F*_2_ value is the highest, it is a CCF; if the *F*_3_ value is the highest, it is a JCF; and if the *F*_4_ value is the highest, it is a TCF. The prediction capacity of the BLDA model was also evaluated by the cross-validated method (leave-one-out)^25^. The correctness values of each discriminant function of RCF, CCF, JCF, and TCF were 97.3%, 100%, 88.5%, 95% in the cross-validation, respectively (Supplemetary Table S7). Most samples were correctly classified. Overall, the canonical discriminant functions can be considered satisfactory in the classification and differentiation of raw and processed CF products.

### Prediction of different types of processed CF products by back-propagation neural network

Compared with linear discriminant analysis, artificial neural network, as a typical nonlinear discriminant, can effectively process sample data with complex information. In this study, BPNN was used to construct a nonlinear prediction model on Matlab R2018b software, which improved the classification result.

In the three-layer network with input, hidden, and output, the training dataset utilised 10 sensors responses (U_1_) as the input layer and 4 predicted groups (RCF, CCF, JCF, TCF) as the output layer. Additionally, the number of neurons in the hidden layer was determined by a series of tests and revisions based on the classic formula: *L* < (m + n)^1/2^ + α, where L is the number of hidden layer nodes, m is the number of input layer nodes, n is the number of output layer nodes, and α is a constant ranging from 1 to 10. Finally, 11 neurons in the hidden layer were found to be sufficient for preferable performance, and more neurons would merely increase training time. A BPNN model with the structure of 10–11–4 was eventually established.

**Figure 4:**
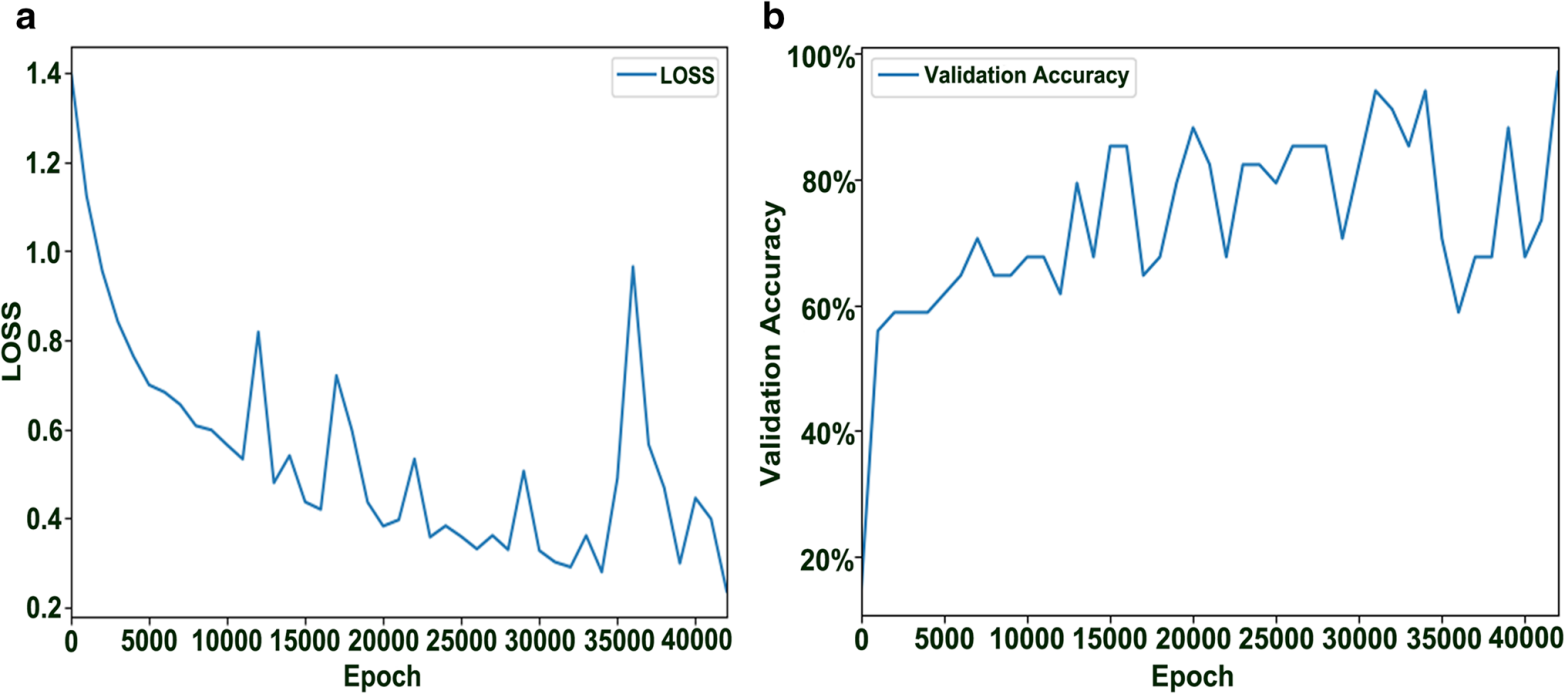
BPNN performance evaluation results of different CF groups. (**a**) Loss value in the training process; (**b**) validation accuracy in the validation process.

Seventy and twenty percent samples were selected randomly as the training and validation sets, respectively, with the remaining samples being used as the test set. The BPNN training process is normally terminated only when the accuracy of the validation set reaches 95% and the total number of training epochs is more than 10,000 times. The BPNN performance evaluation results of different CF groups are shown in Fig. 4. The horizontal axis represents the number of training epochs of the model. The loss value represents the difference between predicted and correct values, and it decreased significantly during the training period of 0–10,000 epochs and then decreased steadily. When the number of training epochs was 42,000, the loss value reached a minimum of 0.2369 (Fig. 4a). Meanwhile, the accuracy of the validation set was 97.06%, and the model training was finished (Fig. 4b). The above results showed that the established BPNN model was qualified^26^. The total correct classification rate of the training and validation sets were 96.83% and 97.06%, respectively. In the test set, the correct classification rate was 93.75% and only one CCF sample was misclassified as an RCF (Supplementary Table S8). In conclusion, BPNN can effectively classify and predict different types of processed CF products.

## Discussion

While optimising the detection conditions for E-nose, most researchers set the incubation temperature above 40 °C^16,17,27^, and our pre-experimental results also showed that the sensor response value would be too low to be detected at low incubation temperatures, hence, the incubation temperature was set at 40–55 °C, which was consistent with that of most previous studies. In addition, we found that among all the factors investigated, injection volume and incubation temperature have the greatest influence on sensor response value. The results indicated that the increase in sensor response values was probably due to the increased volatile components accounted by an increase in injection volume and incubation temperature.

Both SDA and ANOVA were used for sensor optimisation, which has been reported in many previous studies^26,28^. However, to the best of our knowledge, very few studies have compared the two methods. In our study, the SDA was carried out in units of the entire sensor array, while the ANOVA was performed on single sensor. The sensors have no correlation with each other in the ANOVA, which may lead to relatively poor results. This is the biggest difference between the two methods. Therefore, compared with ANOVA, SDA has a greater advantage in sample identification, which was also proved by our experimental results.

DFA is a common analytical method applied in many studies to classify and identify samples using E-nose^29,30^, and most of them are two-dimensional models^18^. However, in our study, we found that the two-dimensional DFA model could not distinguish well among the four processed CF groups, which may be due to the large number of CF groups. Hence, a three-dimensional DFA model can appropriately distinguish between the different CF groups. RCF is close to CCF group, and JCF is close to TCF. The result is consistent with artificial judgement, that is, judging from olfactory senses, RCF odour resembles CCF odour, and that of JCF resembles that of TCF. Furthermore, the digital standard was established for the first time, which realises the objective expression of odour and can be also used to differentiate samples.

The mathematical prediction models of CF products were established by linear discriminant of BLDA and nonlinear discriminant of BPNN. BLDA has been rarely used to classify samples by E-nose, and there is limited research on the formation of discriminant formulas^31^. In our study, odour response-based discriminant formulas were established for the first time, which is very intuitive and easily acceptable because of a clear discriminative coefficient and convenient operation method. Compared with classic BLDA, BPNN is novel and similar to the prediction of human experience owing to its nonlinear characteristics. Similarly, BPNN was used to predict raw and processed CF products by E-nose for the first time. The results showed that the BPNN model has a small loss value and high validation accuracy, indicating that the experimental data fits well. Additionally, the results of recognition and prediction showed that the BPNN of CF samples had a high accuracy rate for training, validation, and test sets. It also indicates that the BPNN has a potential to predict unknown samples. Meanwhile, it was found that the BPNN has a low CCF recognition rate in the test set, which may be due to insufficient sample size. However, based on this small number of training samples, the advantages of artificial neural networks have been highlighted. It is possible that artificial neural network will replace human discrimination in the future.

## Conclusion

The electronic nose of FOX-4000 with 18 different MOS sensors proved to be able to quantify the odour of CF effectively and objectively. In terms of reducing the data dimension and improving classification efficiency, stepwise discriminant in the optimisation of the sensor array had a better performance than one-way ANOVA. Furthermore, based on the optimised array with 10 sensors, the established digital and mode standards can be a tool to control the quality of different types of processed CF products. Although both the discriminant formula and BPNN could predicate different types of processed CF products, BPNN is better to be used to replace human judgement.

## Materials and methods

### Experimental materials

Eighty-eight different samples including 37 batches of RCF, 28 batches of CCF, 13 batches of JCF and 10 batches of TCF samples were collected from their main production locations and identified by Professor Tulin Lu from the Nanjing University of Chinese Medicine (Supplementary Table S9).

### Electronic nose

Odour detection was performed on a commercial FOX-4000 E-nose (Alpha MOS, Toulouse, France), which consists of a sampling apparatus, an array of sensors, an autosampler, air generator equipment and pattern recognition software (Alpha Soft V11.0) for data (Supplementary Fig. S2), and it has a high sensitivity^32^. The sensor array is composed of 18 metal oxide semiconductors (MOS) that are divided into three sets: SET CL2 (LY2/LG, LY2/G, LY2/AA, LY2/GH, LY2/gCT, LY2/gCT), SET A (T30/1, P10/1, P10/2, P40/1, T70/2, PA/2) and SET B (P30/1, P40/2, P30/2, T40/2, T40/1, TA/2). They are placed in three chambers and calibrated regularly in line with the manufacturer’s recommended procedures to ensure stability. The components and main application of sensors are listed in Table 3.

**Table 3:** The components and main application of sensors of FOX-4000.

No.	Name	Description	No.	Name	Description
1	LY2/LG	Oxidizing gas	10	P40/1	Fluorine
2	LY2/G	Ammonia, carbon monoxide	11	T70/2	Aromatic compounds
3	LY2/AA	Ethanol	12	PA/2	Ethanol, ammonia/organic amines
4	LY2/GH	Ammonia/organic amines	13	P30/1	Polar compounds (ethanol)
5	LY2/gCTl	Hydrogen sulfide	14	P40/2	Heteroatom/chloride/aldehydes
6	LY2/gCT	Propane/butane	15	P30/2	Alcohol
7	T30/1	Organic solvents	16	T40/2	Aldehydes
8	P10/1	Hydrocarbons	17	T40/1	Chlorinated compounds
9	P10/2	Methane	18	TA/2	Air quality

### Odour detection and acquisition by Electronic nose

The CF samples were pulverized and sieved (50 mesh). Then the powder was accurately weighed (0.5 g) and transferred to headspace vials (10 mL). After sealing, the vials were loaded into the autosampler. The procedure mainly referred to previous references^33,34^. The time and temperature of headspace incubation were 600 s and 55 °C, respectively. The carrier gas was synthetic dry air with a flow rate of 150 mL/min, and the agitation speed was 500 rpm. Then 1200 µL of the headspace air was automatically injected into the chamber by a syringe at the rate of 500 µL/s. The time of signal acquisition and the time between injections were 120 s and 600 s, respectively. The response values of the 18 sensors of every sample were recorded, and response curves were generated. Samples were analyzed in duplicate (total, 176 sample data).

### Data analysis

Many different multivariate statistical methods were applied in the study. SDA and ANOVA were applied to the sensor array optimisation. LDA was used to confirm the optimisation results of sensors. Percentiles and DFA were also used for establishing digital and mode standard of odour response, respectively. BLDA and BPNN were applied to form two prediction models of different types of processed CF products.

#### Stepwise discriminant analysis

SDA begins with no variables (sensor signals) in the model. The model is examined at each step. If the variable in the model contributes in the least to the discriminatory ability of the model measured by Wilks’ lambda and fails to meet the criterion of keeping, it will be removed. Meanwhile, the new variable is entered. The experiment is terminated after the optimal variable is determined. In this study, SDA was employed to filter out the set of sensors that are most helpful in identifying CF groups.

#### Analysis of variance

ANOVA is a method of portioning variability into identifiable sources of variation and the associated degree of freedom in an experiment^35^. It compares the means of different experimental varieties and determines whether significant differences exist among them^36^. In this study, ANOVA was used to explore whether the response of the sensor contributes significantly to the grouping of the CF samples.

#### Linear discriminant analysis

LDA is a probabilistic parametric classification technique that maximizes the variance between categories and minimizes the variance within categories via data projection from a high-dimensional space to a low-dimensional space^37^. Compared with principal component analysis (PCA), the LDA method can consider not only the similarity of samples but also the category of samples, so as to achieve the maximum differentiation between groups^38^. Here, LDA was used to visualize the classification of samples.

#### Discriminant factorial analysis

DFA is a method to build a visual discriminant model based on known samples. Its modelling process is similar to LDA. However, it can classify a new sample by projecting this sample onto the eigenvectors space and selecting the nearest class^16^. The validation value obtained by cross-validation was used to evaluate the built model.

#### Bayesian linear discriminant analysis

BLDA is a method used to construct multiple discriminant functions for classifying samples by bayesian criteria and to obtain the correct classification rate by training samples with the back generation, in which the ratio of between-class variance is maximized and the within-class variance is minimized^39,40^. It is regarded as an extension of fisher linear discriminant analysis (FLDA) and has shown high performance. Compared with conventional FLDA, the BLDA algorithm employs regularization to avoid overfitting to high dimensional and noisy datasets^41^.

#### Back-propagation neural network

BPNN is one of the most commonly used neural networks and includes input, hidden, and output layers. In the process of training BPNN for analysis, the weights and threshold values of each layer are constantly revised based on the differences between the expected outputs and actual outputs. Thus, a BPNN is a neural network that spreads information in the forward direction and returns the difference in the reverse direction. This training is ceased until the difference between the expected outputs and actual outputs are reduced to a preset range or the scheduled training times are achieved. The prediction model was evaluated by loss value^42^ and validation accuracy^26^.

SDA, ANOVA, LDA and BLDA were performed by SPSS 23.0 (IBM, USA); DFA was performed on Electronic nose software (Alpha Soft V11.0); BPNN was performed by MATLAB R2018b (MathWorks, USA).

## Supplementary Information

Supplementary Information.
