# Supplementary material for: Identification of the raw and processed Crataegi Fructus based on the electronic nose coupled with chemometric methods
Source: Sci Rep. 2021 Jan 20;11:1849. doi: 10.1038/s41598-020-79717-w (PMC7817683; doi:10.1038/s41598-020-79717-w)
Supplement: Supplementary file 1 — Supplementary Information. [file 41598_2020_79717_MOESM1_ESM.docx]

**Supporting Information**

**Identification of the** **raw and processed** ***Crataegi* Fructus based on the electronic nose coupled with** **chemometric methods**

Chenghao FEI^1^, Chenchen REN^1^, Yulin WANG^1^, Lin LI^1^, Weidong LI^1^, Fangzhou YIN^1,^*, Tulin LU^1,^*, and Wu YIN^2,^*

^1^School of pharmacy, Nanjing University of Chinese Medicine, Nanjing, China

^2^State Key Lab of Pharmaceutical Biotechnology, College of Life Sciences, Nanjing University, Nanjing, China

*correspondings: yfz2003@163.com (F. Y.); lutuling2005@126.com (T. L.); wyin@nju.edu.cn (W. Y.)

**

**

**Supplementary Figure 1.** The effects of sample treatment conditions and E-nose detecting parameters on the sensor response

(a, b) Sensor response and RSDs of sensors based on different particle sizes; (c, d) Sensor response and RSDs of sensors based on different sample quality; (e, f) Sensor response and RSDs of sensors based on different headspace incubation time; (g, h) Sensor response and RSDs of sensors based on different headspace incubation temperature; (i, j) Sensor response and RSDs of sensors based on different injected volume.

**
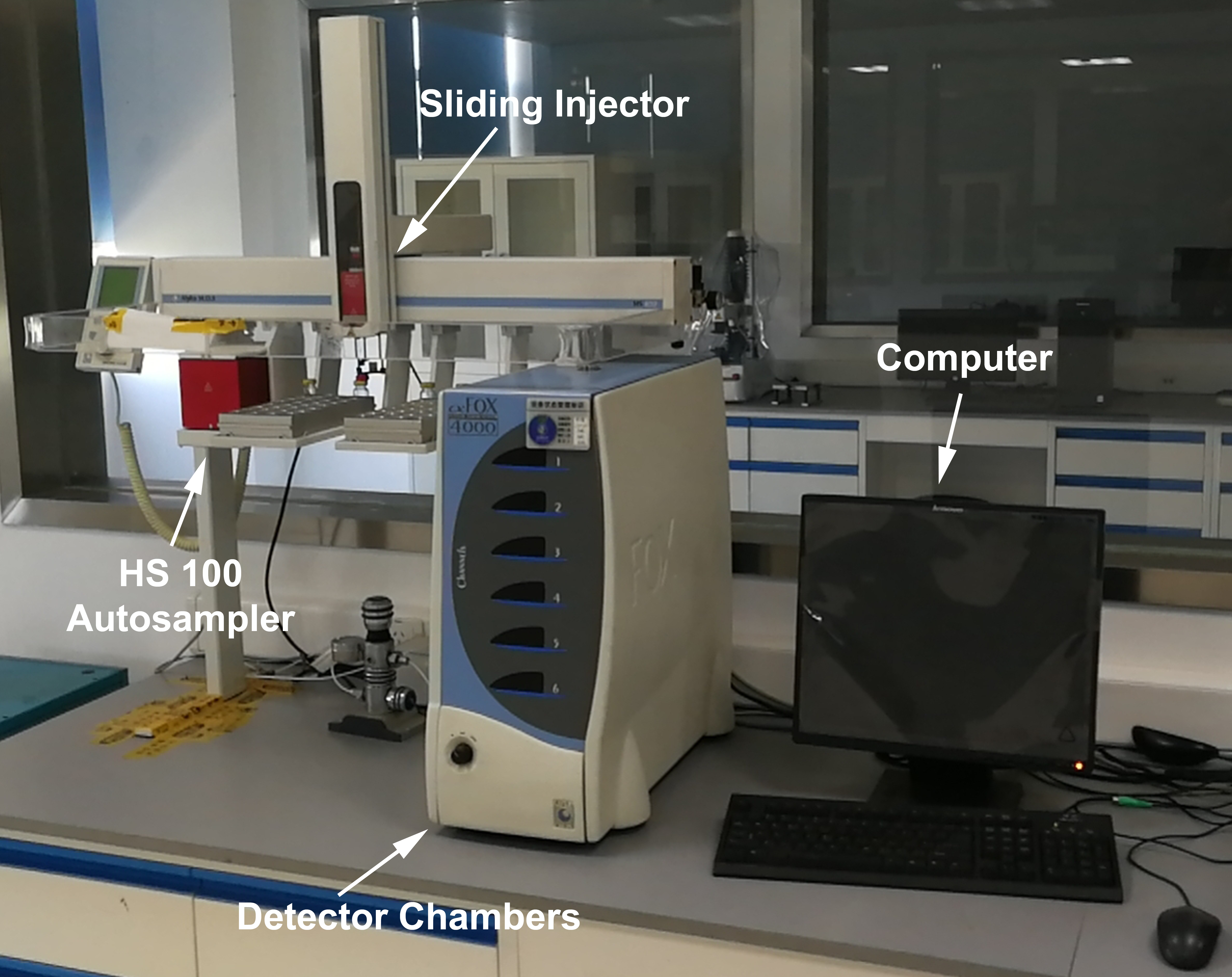
**

**Supplementary Figure 2.** FOX-4000 setup

**Supplementary Table 1.** Results of repeatability for electronic nose detection method

| Sensor | Sensor response | | | | | | RSD (%) |
| --- | --- | --- | --- | --- | --- | --- | --- |
|  | 1 | 2 | 3 | 4 | 5 | 6 |  |
| LY2/LG | 0.070 | 0.069 | 0.071 | 0.068 | 0.068 | 0.073 | 2.556 |
| LY2/G | -0.193 | -0.197 | -0.204 | -0.200 | -0.198 | -0.200 | 1.774 |
| LY2/AA | -0.157 | -0.159 | -0.164 | -0.163 | -0.160 | -0.163 | 1.639 |
| LY2/Gh | -0.283 | -0.289 | -0.300 | -0.294 | -0.292 | -0.295 | 1.850 |
| LY2/gCTl | -0.253 | -0.262 | -0.273 | -0.268 | -0.265 | -0.267 | 2.315 |
| LY2/gCT | -0.048 | -0.049 | -0.050 | -0.050 | -0.050 | -0.051 | 2.037 |
| T30/1 | 0.562 | 0.570 | 0.584 | 0.577 | 0.575 | 0.578 | 1.197 |
| P10/1 | 0.737 | 0.745 | 0.756 | 0.753 | 0.751 | 0.751 | 0.810 |
| P10/2 | 0.517 | 0.520 | 0.525 | 0.527 | 0.524 | 0.523 | 0.629 |
| P40/1 | 0.637 | 0.643 | 0.652 | 0.650 | 0.648 | 0.647 | 0.781 |
| T70/2 | 0.555 | 0.562 | 0.577 | 0.570 | 0.568 | 0.571 | 1.251 |
| PA/2 | 0.723 | 0.732 | 0.745 | 0.740 | 0.738 | 0.738 | 0.957 |
| P30/1 | 0.765 | 0.771 | 0.782 | 0.777 | 0.775 | 0.778 | 0.688 |
| P40/2 | 0.688 | 0.691 | 0.699 | 0.695 | 0.693 | 0.696 | 0.477 |
| P30/2 | 0.805 | 0.804 | 0.812 | 0.806 | 0.805 | 0.812 | 0.410 |
| T40/2 | 0.374 | 0.377 | 0.382 | 0.381 | 0.380 | 0.380 | 0.783 |
| T40/1 | 0.424 | 0.424 | 0.425 | 0.432 | 0.429 | 0.424 | 0.734 |
| TA/2 | 0.511 | 0.514 | 0.520 | 0.522 | 0.520 | 0.516 | 0.764 |

**Supplementary Table 2.** Result of stability for electronic nose detection method

| Sensor | Sensor response | | | | | | | | RSD（%） |
| --- | --- | --- | --- | --- | --- | --- | --- | --- | --- |
|  | 0 h | 2 h | 4 h | 6 h | 8 h | 10 h | 14h | 16h |  |
| LY2/LG | 0.070 | 0.069 | 0.070 | 0.069 | 0.070 | 0.072 | 0.074 | 0.074 | 2.502 |
| LY2/G | -0.198 | -0.190 | -0.193 | -0.193 | -0.196 | -0.197 | -0.205 | -0.201 | 2.284 |
| LY2/AA | -0.160 | -0.154 | -0.157 | -0.157 | -0.159 | -0.159 | -0.168 | -0.164 | 2.563 |
| LY2/Gh | -0.290 | -0.278 | -0.283 | -0.283 | -0.288 | -0.288 | -0.304 | -0.297 | 2.698 |
| LY2/gCTl | -0.263 | -0.251 | -0.255 | -0.256 | -0.261 | -0.261 | -0.277 | -0.271 | 3.124 |
| LY2/gCT | -0.049 | -0.048 | -0.048 | -0.048 | -0.049 | -0.049 | -0.050 | -0.049 | 1.408 |
| T30/1 | 0.572 | 0.559 | 0.566 | 0.564 | 0.571 | 0.572 | 0.589 | 0.579 | 1.514 |
| P10/1 | 0.746 | 0.736 | 0.740 | 0.740 | 0.744 | 0.746 | 0.761 | 0.752 | 1.015 |
| P10/2 | 0.521 | 0.515 | 0.516 | 0.515 | 0.517 | 0.519 | 0.534 | 0.525 | 1.193 |
| P40/1 | 0.644 | 0.635 | 0.640 | 0.639 | 0.643 | 0.645 | 0.657 | 0.649 | 1.006 |
| T70/2 | 0.565 | 0.552 | 0.558 | 0.558 | 0.564 | 0.564 | 0.583 | 0.573 | 1.624 |
| PA/2 | 0.733 | 0.721 | 0.728 | 0.727 | 0.732 | 0.734 | 0.754 | 0.744 | 1.332 |
| P30/1 | 0.773 | 0.763 | 0.768 | 0.767 | 0.772 | 0.772 | 0.789 | 0.782 | 0.997 |
| P40/2 | 0.693 | 0.686 | 0.691 | 0.690 | 0.694 | 0.695 | 0.704 | 0.700 | 0.767 |
| P30/2 | 0.807 | 0.800 | 0.805 | 0.804 | 0.808 | 0.808 | 0.816 | 0.811 | 0.546 |
| T40/2 | 0.378 | 0.372 | 0.375 | 0.374 | 0.378 | 0.379 | 0.389 | 0.383 | 1.331 |
| T40/1 | 0.425 | 0.423 | 0.427 | 0.426 | 0.426 | 0.432 | 0.437 | 0.430 | 1.023 |
| TA/2 | 0.515 | 0.509 | 0.514 | 0.513 | 0.516 | 0.520 | 0.529 | 0.521 | 1.128 |

**Supplementary Table 3.** Result of stepwise discriminant based on 18 sensors

| Step | Entered | Wilks’ Lambda | | | | | | | | | | | |
| --- | --- | --- | --- | --- | --- | --- | --- | --- | --- | --- | --- | --- | --- |
|  |  | Lambda | *df*1 | *df*2 | *df*3 | Exact *F* | | | | Approximate *F* | | | |
|  |  |  |  |  |  | Statistic | *df*1 | *df*2 | *p* | Statistic | *df*1 | *df*2 | *p* |
| 1 | P30/1 | 0.411 | 1 | 3 | 172.000 | 82.285 | 3 | 172.000 | 0.000 |  |  |  |  |
| 2 | P30/2 | 0.057 | 2 | 3 | 172.000 | 182.279 | 6 | 342.000 | 0.000 |  |  |  |  |
| 3 | P10/2 | 0.040 | 3 | 3 | 172.000 |  |  |  |  | 127.180 | 9 | 413.886 | 0.000 |
| 4 | P40/2 | 0.009 | 4 | 3 | 172.000 |  |  |  |  | 181.457 | 12 | 447.423 | 0.000 |
| 5 | PA/2 | 0.006 | 5 | 3 | 172.000 |  |  |  |  | 169.733 | 15 | 464.175 | 0.000 |
| 6 | T40/2 | 0.002 | 6 | 3 | 172.000 |  |  |  |  | 200.159 | 18 | 472.833 | 0.000 |
| 7 | T40/1 | 0.002 | 7 | 3 | 172.000 |  |  |  |  | 187.046 | 21 | 477.212 | 0.000 |
| 8 | LY2/AA | 0.001 | 8 | 3 | 172.000 |  |  |  |  | 175.828 | 24 | 479.151 | 0.000 |
| 9 | T30/1 | 0.001 | 9 | 3 | 172.000 |  |  |  |  | 168.436 | 27 | 479.607 | 0.000 |
| 10 | LY2/gCTl | 0.001 | 10 | 3 | 172.000 |  |  |  |  | 159.048 | 30 | 479.113 | 0.000 |

Wilks’ lambda is a measure which each function separates cases into groups.

At each step, the sensor that minimizes the overall Wilks’ Lambda is entered.

In *F* test, minimum partial *F* entered model is 3.84; maximum partial *F* removed is 2.71. Both the *F* value and *p* value are used to evaluate the significance of the sensor for discrimination.

**Supplementary Table 4.** Result of one-way ANOVA based on 18 sensors

| Sensor | Between Groups | | Within Groups |
| --- | --- | --- | --- |
|  | *F* | *p* | Mean Square (×10^-3^) |
| LY2/LG | 2.477 | 0.063 | 3.07 |
| LY2/G* | 3.029 | 0.031 | 2.53 |
| LY2/AA* | 3.071 | 0.029 | 1.32 |
| LY2/Gh* | 3.686 | 0.013 | 5.89 |
| LY2/gCTl* | 2.947 | 0.034 | 4.34 |
| LY2/gCT* | 5.155 | 0.002 | 0.18 |
| T30/1* | 33.254 | 0.000 | 6.18 |
| P10/1* | 19.163 | 0.000 | 4.08 |
| P10/2* | 6.126 | 0.001 | 1.53 |
| P40/1* | 11.229 | 0.000 | 2.54 |
| T70/2* | 22.727 | 0.000 | 6.71 |
| PA/2* | 32.692 | 0.000 | 4.14 |
| P30/1* | 82.285 | 0.000 | 2.23 |
| P40/2* | 39.319 | 0.000 | 2.59 |
| P30/2* | 20.059 | 0.000 | 3.88 |
| T40/2* | 6.878 | 0.000 | 1.70 |
| T40/1 | 0.961 | 0.412 | 0.79 |
| TA/2* | 7.457 | 0.000 | 2.75 |

**p* ＜ 0.05.

“Between Groups” means the data among the four CF sample groups.

“Within Groups” means the data among the 18 sensors of each CF group.

**Supplementary Table 5.** Result of the normal distribution test based on the sensor array U_1_

| Sensor | *p* value | | | |
| --- | --- | --- | --- | --- |
|  | RCF | CCF | JCF | TCF |
| LY2/AA | 0.000* | 0.000* | 0.249 | 0.011* |
| LY2/gCTl | 0.000* | 0.000* | 0.116 | 0.012* |
| T30/1 | 0.066 | 0.001* | 0.366 | 0.064 |
| P10/2 | 0.002* | 0.000* | 0.171 | 0.046* |
| PA/2 | 0.200 | 0.042* | 0.221 | 0.071 |
| P30/1 | 0.200 | 0.024* | 0.664 | 0.061 |
| P40/2 | 0.016* | 0.000* | 0.328 | 0.069 |
| P30/2 | 0.200 | 0.030* | 0.191 | 0.042* |
| T40/2 | 0.054 | 0.001* | 0.278 | 0.086 |
| T40/1 | 0.000* | 0.000* | 0.356 | 0.014* |

**p* ＜ 0.05.

**Supplementary Table 6.** Results of the nonparametric test of reference range based on the sensor array U_1`_

| Test method | Test variable | LY2/AA | LY2/gCTl | T30/1 | P10/2 | PA/2 | P30/1 | P40/2 | P30/2 | T40/2 | T40/1 |
| --- | --- | --- | --- | --- | --- | --- | --- | --- | --- | --- | --- |
| Kruskal-Wallis H Test | Chi-Square | 14.294 | 13.954 | 65.239 | 23.959 | 62.300 | 79.933 | 65.946 | 46.744 | 24.699 | 13.356 |
|  | df | 3 | 3 | 3 | 3 | 3 | 3 | 3 | 3 | 3 | 3 |
|  | Asymp. Sig. | 0.003 | 0.003 | 0.000 | 0.000 | 0.000 | 0.000 | 0.000 | 0.000 | 0.000 | 0.004 |
| Median Test | N | 176 | 176 | 176 | 176 | 176 | 176 | 176 | 176 | 176 | 176 |
|  | Median | -0.103 | -0.153 | 0.530 | 0.388 | 0.557 | 0.720 | 0.675 | 0.760 | 0.324 | 0.224 |
|  | Chi-Square | 17.546 | 15.470 | 51.464 | 21.633 | 45.599 | 43.223 | 48.999 | 39.561 | 18.773 | 10.787 |
|  | df | 3 | 3 | 3 | 3 | 3 | 3 | 3 | 3 | 3 | 3 |
|  | Asymp. Sig. | 0.001 | 0.001 | 0.000 | 0.000 | 0.000 | 0.000 | 0.000 | 0.000 | 0.000 | 0.013 |

**Supplementary Table 7.** Cross-validation results by BLDA on CF samples

|  |  | Predicted Group Membership | | | |  |
| --- | --- | --- | --- | --- | --- | --- |
|  | Group | 1 | 2 | 3 | 4 | Total |
| Count | 1 | 72 | 2 | 0 | 0 | 74 |
|  | 2 | 0 | 56 | 0 | 0 | 56 |
|  | 3 | 0 | 0 | 23 | 3 | 26 |
|  | 4 | 0 | 0 | 1 | 19 | 20 |
| % | 1 | 97.3 | 2.7 | 0.0 | 0.0 | 100.0 |
|  | 2 | 0.0 | 100.0 | 0.0 | 0.0 | 100.0 |
|  | 3 | 0.0 | 0.0 | 88.5 | 11.5 | 100.0 |
|  | 4 | 0.0 | 0.0 | 5.0 | 95.0 | 100.0 |

1, 2, 3, 4 in column: the sample actually belonged to RCF, CCF, JCF, TCF, respectively.

1, 2, 3, 4 in row: the sample was predicted to RCF, CCF, JCF, TCF, respectively.

**Supplementary Table 8.** Prediction results of CF samples in test set by BPNN

|  |  | Predicted group | | | | |
| --- | --- | --- | --- | --- | --- | --- |
|  |  | RCF | CCF | JCF | TCF |  |
| Actual group | RCF | 7 | 0 | 0 | 0 |  |
|  | CCF | 1 | 4 | 0 | 0 |  |
|  | JCF | 0 | 0 | 2 | 0 |  |
|  | TCF | 0 | 0 | 0 | 2 |  |

**Supplementary Table 9.** Information of all batches of CF samples

| RCF | NO. | Origin | Batch ID | NO. | Origin | Batch ID | NO. | Origin | Batch ID | NO. | Origin | Batch ID |
| --- | --- | --- | --- | --- | --- | --- | --- | --- | --- | --- | --- | --- |
|  | 1 | Shandong | 151016 | 2 | Shandong | 151204 | 3 | Anhui | 160535 | 4 | Shandong | 160310 |
|  | 5 | Shandong | 160413 | 6 | Shandong | 160301 | 7 | Shandong | 160102 | 8 | Hebei | 160301 |
|  | 9 | Shandong | 160503 | 10 | Shandong | 160312 | 11 | Shandong | 150106 | 12 | Shandong | 160112 |
|  | 13 | Shandong | 160308 | 14 | Shandong | 160301 | 15 | Shandong | 160507 | 16 | Hebei | 160208 |
|  | 17 | Hebei | 160519 | 18 | Hebei | 160116 | 19 | Shandong | 160513 | 20 | Shandong | 160201 |
|  | 21 | Jiangsu | 151027 | 22 | Shandong | 160101 | 23 | Henan | 150805 | 24 | Henan | 160403 |
|  | 25 | Shandong | 151102 | 26 | Shandong | 150901 | 27 | Shandong | 160312 | 28 | Shandong | 160401 |
|  | 29 | Shandong | 160413 | 30 | Shandong | 151105 | 31 | Hebei | 150910 | 32 | Shandong | 160415 |
|  | 33 | Henan | 160511 | 34 | Henan | 160608 | 35 | Shandong | 160624 | 36 | Shandong | 160520 |
|  | 37 | Shandong | 160807 |  |  |  |  |  |  |  |  |  |
| CCF | NO. | Origin | Batch ID | NO. | Origin | Batch ID | NO. | Origin | Batch ID | NO. | Origin | Batch ID |
|  | 1 | Anhui | 160535 | 2 | Anhui | 160416 | 3 | Shandong | 160310 | 4 | Shandong | 160212 |
|  | 5 | Shandong | 160503 | 6 | Shandong | 160529 | 7 | Shandong | 150106 | 8 | Shandong | 160607 |
|  | 9 | Shandong | 160301 | 10 | Shandong | 160510 | 11 | Shandong | 160401 | 12 | Hebei | 160208 |
|  | 13 | Hebei | 160315 | 14 | Shandong | 160513 | 15 | Shandong | 160501 | 16 | Shandong | 160702 |
|  | 17 | Hebei | 160222 | 18 | Shandong | 160604 | 19 | Shandong | 160716 | 20 | Henan | 160710 |
|  | 21 | Henan | 160613 | 22 | Shandong | 160722 | 23 | Shandong | 160720 | 24 | Shandong | 160722 |
|  | 25 | Hebei | 160724 | 26 | Henan | 160628 | 27 | Henan | 160524 | 28 | Shandong | 160606 |
| JCF | NO. | Origin | Batch ID | NO. | Origin | Batch ID | NO. | Origin | Batch ID | NO. | Origin | Batch ID |
|  | 1 | Shandong | 150629 | 2 | Shandong | 160325 | 3 | Shandong | 151129 | 4 | Shandong | 160419 |
|  | 5 | Shandong | 160325 | 6 | Hebei | 151202 | 7 | Shandong | 160508 | 8 | Shandong | 160325 |
|  | 9 | Henan | 160511 | 10 | Shandong | 160624 | 11 | Shandong | 160606 | 12 | Shandong | 160725 |
|  | 13 | Shandong | 160628 |  |  |  |  |  |  |  |  |  |
| TCF | NO. | Origin | Batch ID | NO. | Origin | Batch ID | NO. | Origin | Batch ID | NO. | Origin | Batch ID |
|  | 1 | Henan | 160710 | 2 | Shandong | 160401 | 3 | Shandong | 160801 | 4 | Shandong | 160722 |
|  | 5 | Shandong | 160606 | 6 | Shandong | 160722 | 7 | Shandong | 160724 | 8 | Shandong | 160616 |
|  | 9 | Hebei | 160701 | 10 | Shandong | 151029 |  |  |  |  |  |  |
